# Fate and future of the medical students in Ukraine: A silently bubbling educational crisis

**DOI:** 10.1111/medu.14818

**Published:** 2022-05-18

**Authors:** Nityanand Jain, Deepkanwar Singh Panag, Mahek Srivastava, Srinithi Mohan, Swarali Yatin Chodnekar, Amir Reza Akbari, Divyam Raj, Dionysia Kravarioti, Dorota Świątek, Emilia Platos, Ketevani Khetsuriani, Kitija Lucija Gristina, Kyriacos Evangelou, Marinela Lica, Revathi Sairaj Thirumushi, Sofia Rozani, Shivani Jain, Pavlo Fedirko, Aigars Reinis, Mara Pilmane

**Affiliations:** ^1^ Faculty of Medicine Riga Stradiņš University Riga Latvia; ^2^ Faculty of Medicine Charles University Hradec Králové Czechia; ^3^ Faculty of Medicine Teaching University Geomedi LLC Tbilisi Georgia; ^4^ King's Mill Hospital Sherwood Forest Hospitals NHS Foundation Trust Nottinghamshire UK; ^5^ Department of Medicine Democritus University of Thrace Alexandroupolis Greece; ^6^ Faculty of Medicine Medical University of Lodz Lodz Poland; ^7^ Faculty of Medicine Medical University of Warsaw Warsaw Poland; ^8^ Faculty of Medicine David Tvildiani Medical University Tbilisi Georgia; ^9^ Department of Medicine National and Kapodistrian University of Athens Athens Greece; ^10^ Regional Hospital Fier Fier Albania; ^11^ Department of Oral and Maxillofacial Surgery Genesis Institute of Dental Sciences and Research Ferozepur Punjab India; ^12^ Institute of Radiation Hygiene and Epidemiology State Institution ‐ National Research Center for Radiation Medicine of the National Academy of Medical Sciences of Ukraine Kyiv Ukraine; ^13^ Department of Biology and Microbiology Riga Stradiņš University Riga Latvia; ^14^ Department of Morphology Institute of Anatomy and Anthropology Riga Latvia

## Abstract

Jain et al. reflect on the Russia‐Ukraine war and argue that although there is a broad consensus on the need for intervention, focus should be on providing immediate accommodative measures.

In a recently published scoping review in *Medical Education*, Dobiesz et al.^1^ discuss the nature and extent of the impact of wars on the disruption of medical education in conflict zones. The authors systematically and thematically highlight five different categories of barriers and related interventions which were found to be common during all conflicts irrespective of the period in history. These categories included challenges with the curriculum, manpower, wellness of educator and students, resources and oversight of quality of education.

This insightful review could not have been more timely given the increasing polarisation, radicalisation and instigation across the globe. According to the Armed Conflict Location & Event Data Project (ACLED), since the beginning of the Covid‐19 pandemic, more than 77 000 armed engagements have occurred across different regions of the world.^2^ Whilst most of these engagements were at the local level, some of them have stretched for years, have been deadlier and have claimed tens of thousands of civilian lives. With this commentary, we would like to further the discussion presented by Dobiesz et al.^1^ in the context of the ongoing Russo‐Ukrainian war. We aim to focus more on the toll that this war has taken and will take on the international medical students enrolled in Ukrainian universities, challenges faced by students seeking transfer in neighbouring EU countries and the obstacles in their smooth integration.

Since the beginning of the Covid‐19 pandemic, more than 77 000 armed engagements have occurred across different regions of the world.

Ukraine has over the years become a popular student‐friendly destination for medical education. Its age‐old academic experiences, availability of infrastructure and provision of easily accessible value‐for‐money educational opportunities helped it achieve the status of a global educational hub. Around 80 000 foreign students were estimated to be enrolled in various undergraduate and postgraduate study courses as of late 2020, with medicine being the most popular study field. Students from India, Morocco, Turkmenistan, Azerbaijan, Nigeria and Turkey represented the largest international student communities in Ukraine.

The rapid turn of events left medical students in the middle of an armed conflict without the chance of being able to leave. Although public advisories were issued by foreign embassies in the country, the Ukrainian government and universities underplayed the possibility of an invasion.^3^ This created a huge dilemma for the students on whether to leave the country or not. Whilst there were many students who proactively returned to their home countries or never came back due to Covid‐19 restrictions, others who stayed back got caught in a mental, physical, financial and emotional storm. The students had to rush out to safe locations, underground bunkers, and subway stations. Some even travelled (and walked) miles overnight to reach the capital Kyiv or crossed the border into the neighbouring EU countries. Most students left with whatever they felt was essential for survival, leaving behind their educational documents, resident permits, and other important identifications.

Since then, the future of these students has been uncertain as they look for opportunities for transfers in their home countries or abroad. Multiple EU universities have announced plans to enrol medical students fleeing Ukraine along with provision of accommodation, fee waivers, and psychological support for the students and family members. However, most of these opportunities have been prioritized for Ukrainian nationals, whilst international students have been asked to register for waiting lists. Even for local Ukrainian students, lack of financial resources to resume education and sustain in a foreign nation; differences in attitudes and beliefs; experiences of discrimination from peers, educators and community members; socio‐emotional and mental health issues; issues with overcoming interrupted schooling and intercultural competencies; learning a new language of instruction; marginalisation and adjusting to an unfamiliar education system pose significant challenges.

However, most of these opportunities have been prioritized for Ukrainian nationals, whilst international students have been asked to register for waiting lists.

Back in the home countries, the situation has been equally unsupportive. In Nigeria, for example, there are ongoing strikes of academic staff in higher education universities, which has forced the students to look for other opportunities.^4^ Other countries like Israel, Turkey and India have reported provisions for accommodation; however, regulations regarding the same have not been released or remain ambiguous. After intense international pressure, some Ukrainian universities resumed online education, yet there remain concerns regarding the gaps in the education and the deterioration in the quality of education. Even before the invasion, Covid‐19 pandemic had led to multiple challenges for the medical students. There were difficulties developing practical skills especially in clinical subjects like surgery and infectious diseases, with some surveys describing remote learning experience at Ukrainian universities as tiring, ineffective and dull.^5^ Several technical challenges were highlighted by students in remote cities regarding poor internet connections, outages and their inability to obtain technical equipment.^5^ Another challenge highlighted was with some professors having limited expertise in digital proficiency. Recent reports have indicated towards attacks and bombing on schools and universities, which have caused infrastructural damage to the premises, thereby delaying the resumption of educational processes.

Recent reports have indicated towards attacks and bombing on schools and universities, which have caused infrastructural damage to the premises, thereby delaying the resumption of educational processes.

Another important associated hurdle is the oversight issue, that is, hurdles associated with differences in the accreditation, governance and certification processes.^1^ Even though Ukraine entred the Bologna Process in 2005 and passed resolution for implementation of the Unified State Qualification Exam (USQE) in 2018, the reforms have been slow in implementation and adoption.^6^, ^7^ According to the Ministry of Health of Ukraine, it had very limited objective data regarding the current quality of medical education in the country and hence could not formulate a more targeted state policy.^6^ These differences make it difficult for the host and home countries to smoothly integrate the students. Additionally, resistance from already enrolled students against such integration has been also reported in many countries.^8^

Another important associated hurdle is the oversight issue, that is, hurdles associated with differences in the accreditation.

In wartimes, doctors and medical staff have been subject to irregular and targeted violence and kidnapping, which forces a mass exodus of medical staff. This forced displaced leads to a drain in the medical personnel supply and the local education system that will train the future healthcare providers. Moreover, there has been a shift in the way that hospitals have become trauma and emergency treatment centres at the expense of primary healthcare and essential services. Naturally, there are no active teaching hospitals left. Then there are concerns regarding the diversion of medical students and staff from their studies to work on the frontline, which can translate to indefinite hiatus in their education.^9^ In general, it gives rise to multiple other issues regarding ethics, legality and personal wellbeing of students and educators.

However, a semi‐structured interview with medical students who experienced the 2006 Lebanon‐Israel war revealed that the students view their wartime experience positively.^10^ The students described their experience as resourceful, gratifying, and educational in terms of exposure to normally not exposed pathologies.^10^ Mclean et al.^11^ further this argument by suggesting that such situations test and help in developing the reflective skills and higher professionalism of the medical students and health professionals that can aid in maintaining moral purpose in difficult situations like wars and pandemics. Perhaps, medical students who have volunteered to stay back could be given field and practical trainings whilst serving on the frontlines in more relevant fields of trauma, orthopaedics, emergency medicine, infectious diseases, and so forth, which would help bridging the educational gaps.

In the short term, online courses from partner universities, voluntary participation of university professors in evening school programmes, bridging courses and sending tangible educational material like books, notes and models could be done. Basic language courses in host countries, virtual provision of psychological support, establishment of short‐term qualification equivalency, issuance of temporary education documents, crediting course points and free access to scientific literature for affected students should be encouraged. Many educational companies like Elsevier are offering free access to ClinicalKey, Complete Anatomy, and Osmosis, thereby enabling medical students and professionals in Ukraine to access all necessary information. The European Students Union (ESU) has announced an emergency hotline for students wishing to leave Ukraine. Additionally, ESU and its partners have compiled files with useful information on border crossing, safety/security tips and a guide for refugees in Russian, English and Ukrainian.

In the longer run, there is need to work on quality assurances to comply with the European Higher Education Area (EHEA), along with reconciling the gap between the ECTS system and the current credit system in Ukraine. The focus needs to shift more towards modernization of the curriculum and pedagogical methods. The DECIDE Swiss‐Ukrainian project (decentralisation for improved democratic education) is a nudge in the right direction as it helps demonstrate how to strengthen the capacity of decentralised management. Continuation of the short‐term measures until an ordered transition to a better‐established education system is complete is needed.

Continuation of the short‐term measures until an ordered transition to a better‐established education system is complete is needed.

There is a consensus that there is a lot to be done—both immediately and in the long run. Although broader questions like which university will award the final degree and where would it be recognised, where would students be able to practise remains to be answered, more important is to focus on what steps need to be taken to reach the point where students can be awarded a degree or be allowed to practise medicine in the country they choose.
